# Estimating the proportion of nonsense variants undergoing the newly described phenomenon of manufactured splice rescue

**DOI:** 10.1038/s41431-023-01495-6

**Published:** 2023-11-27

**Authors:** Bushra Haque, David Cheerie, Saba Birkadze, Alice Linyan Xu, Thomas Nalpathamkalam, Bhooma Thiruvahindrapuram, Susan Walker, Gregory Costain

**Affiliations:** 1 Program in Genetics & Genome Biology, SickKids Research Institute, Toronto, ON Canada; 2 Department of Molecular Genetics, University of Toronto, Toronto, ON Canada; 3 The Centre for Applied Genomics (TCAG), SickKids Research Institute, Toronto, ON Canada; 4 Division of Clinical & Metabolic Genetics, The Hospital for Sick Children (SickKids), and Department of Paediatrics, University of Toronto, Toronto, ON Canada

**Keywords:** Genetics research, Medical genetics

## Abstract

A recent report described a nonsense variant simultaneously creating a donor splice site, resulting in a truncated but functional protein. To explore the generalizability of this unique mechanism, we annotated >115,000 nonsense variants using SpliceAI. Between 0.61% (donor gain delta score >0.8, for high precision) and 2.57% (>0.2, for high sensitivity) of nonsense variants were predicted to create new donor splice sites at or upstream of the stop codon. These variants were less likely than other nonsense variants in the same genes to be classified as pathogenic/likely pathogenic in ClinVar (*p* < 0.001). Up to 1 in 175 nonsense variants were predicted to result in small in-frame deletions and loss-of-function evasion through this “manufactured splice rescue” mechanism. We urge caution when interpreting nonsense variants where manufactured splice rescue is a strong possibility and correlation with phenotype is challenging, as will often be the case with secondary findings and newborn genomic screening programs.

## Introduction

**Fig. 1:**
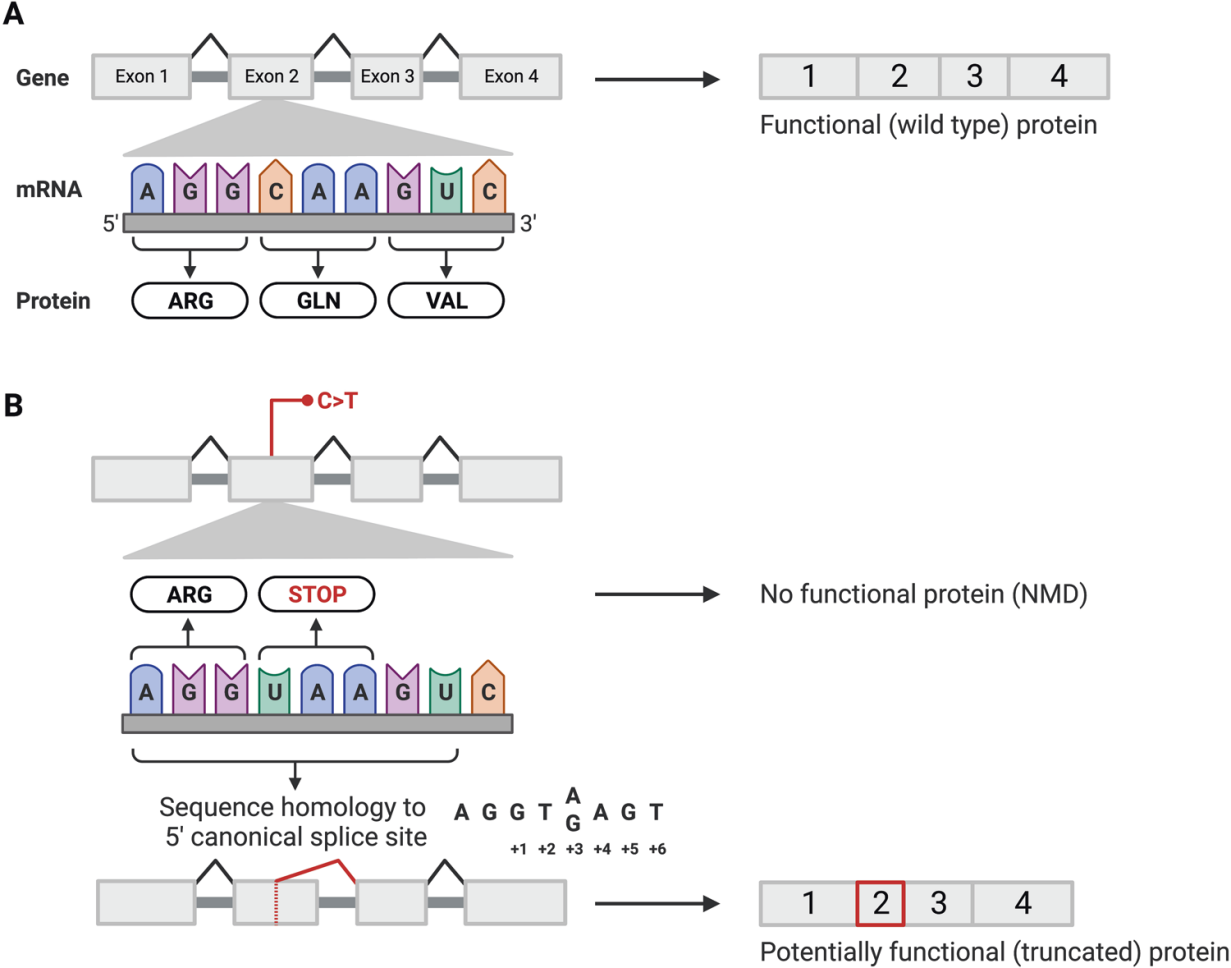
Diagram of proposed mechanism by which a nonsense variant could result in aberrant splicing and thus a potentially functional protein product. **A** Grossly simplified depiction of the “normal” splicing of a 4-exon protein coding gene. **B** Example of a sequence variant that could simultaneously result in a stop-gain and in activation of a cryptic 5’ (donor) splice site. If use of the latter results in a small in-frame deletion, there may be a truncated but functional protein product. In the example shown, the pre-mRNA position of the new splice site would be 2 nucleotides upstream of the variant (i.e., delta position = −2). Created with BioRender.com.

Stop-gain (nonsense) variants are typically assumed to result in loss-of-function, and assigned “very strong” evidence in favour of pathogenicity [1, 2]. A recent report described a nonsense variant in *BUD13* [NM_032725.4:c.688C>T; p.(Arg230*)] that simultaneously activated a new cryptic donor splice site in the same canonical isoform [3]. Surprisingly, the alternative splice product resulted in a truncated but functional protein product, and converted a loss-of-function into a hypomorphic allele [3]. Intrafamilial phenotypic severity of the associated progressive multisystem disease was correlated with the expression level of the truncated protein [3]. This molecular mechanism, which we will term “manufactured splice rescue”, is distinct from nonsense-associated altered splicing (NAS) [4–6], and is not acknowledged in variant interpretation guidelines [1, 2, 7]. The nucleotide triplets TAA and TGA are both stop codons and highly conserved components of canonical splice sites (+2 to +4 positions; Fig. 1), meaning that these codons may be susceptible to cryptic splicing effects. The prevalence of nonsense variants potentially triggering manufactured splice rescue is unknown. We describe the predicted splicing effects of >115,000 single nucleotide nonsense variants, finding that ~1 in 40 variants (2.57%) potentially create new donor splice sites and that ~1 in 175 variants might result in small in-frame deletions rather than a definite loss-of-function.

## Methods

To investigate the generalizability of this “manufactured splice rescue” phenomenon, we used advanced in silico methods and large datasets. We extracted single nucleotide nonsense variants from three variant databases: gnomAD (v3.1.2 and v2.1.1) [8], ClinVar (download date: August 29, 2022) [9], and MSSNG, the largest genome sequencing database for autism with deep phenotyping (latest release: October 16, 2019) [10]. We restricted to canonical transcripts of protein-coding genes, and excluded nonsense variants in the last exon, as these would already be treated cautiously in their interpretation [1, 2]. The remaining 115,171 unique variants (gnomAD: *n* = 84,891; ClinVar: *n* = 33,517; MSSNG: *n* = 5904) were then annotated with SpliceAI using Ensembl Variant Effect Predictor and/or a custom script developed at The Centre for Applied Genomics (TCAG) [11, 12]. We used author-recommended cutoffs for SpliceAI donor gain (DG) delta scores: ≥0.2 (high recall), ≥0.5, and ≥0.8 (high precision) [11]. Recognizing that predicted splicing changes downstream to the variant stop codon would not prevent nonsense mediated decay (NMD), we considered only those variants with DG scores meeting pre-set cutoffs that also had (strand-corrected) pre-mRNA positions/delta positions [11] <3 as potentially resulting in manufactured splice rescue. We used Alamut Visual Plus (v1.7, © 2022 SOPHiA) to inspect the predicted splicing impact of a subset of variants using additional in silico tools [13]. Whether a partial exon deletion resulting from mis-splicing would be in-frame or out-of-frame was based on the difference between the DG position and the exon end position (determined using ExonCalculator; github.com/haqueb2/ExonCalculator). We considered in-frame deletions of less than 10% of the coding transcript to be those potentially resulting in loss-of-function evasion [7]. Protein domains were annotated using InterPro domains [14] with ANNOVAR. Statistical analyses, including Chi-squared, Mann–Whitney *U*, and Wilcoxon Rank-Sum tests, were performed using R statistical software, version 4.1.0 (R Foundation for Statistical Computing) with two-tailed statistical significance set at *p* < 0.05.

## Results

**Fig. 2:**
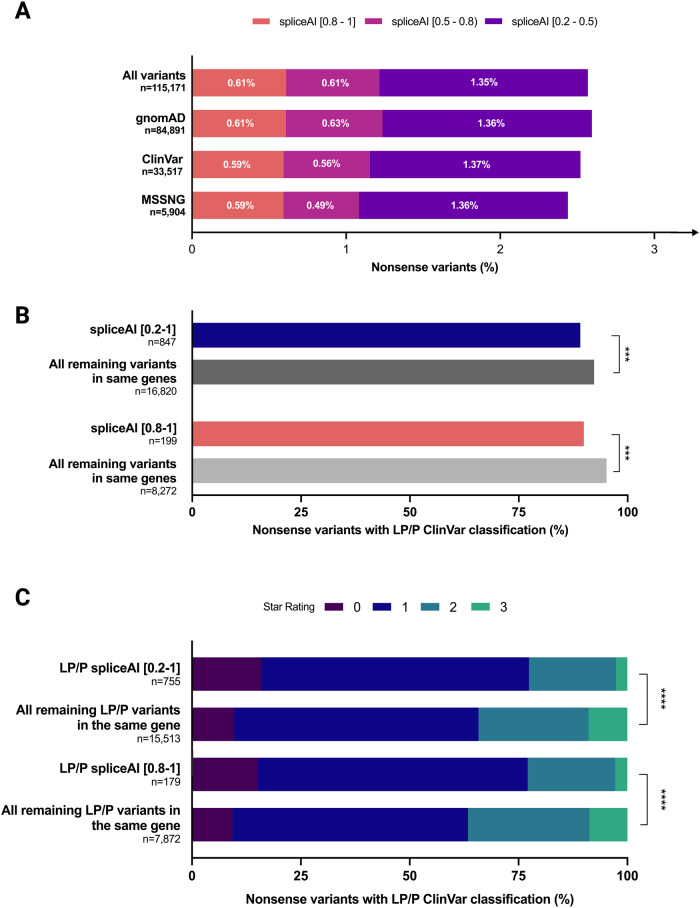
A consistent proportion of nonsense variants across large-scale databases may create new donor splice sites. **A** Stacked bar chart with percentage of nonsense variants predicted to create donor gain sites using SpliceAI. Nonsense variants from three variant databases (gnomAD [8], ClinVar [9], MSSNG [10]) were annotated with DG SpliceAI scores and categorized into three score categories: [0.8–1], [0.5–0.8), and [0.2–0.5) (see Methods for additional details). **B** Bar chart including percentages of likely pathogenic/pathogenic (LP/P) nonsense variants in ClinVar with SpliceAI scores ≥0.2 or ≥0.8, compared to all other variants in the same genes. **C** Stacked bar chart with percentages of ClinVar “star ratings” for LP/P variants with SpliceAI scores ≥0.2 or ≥0.8, compared to all other variants in the same genes. Wilcoxon Rank-Sum test was used to evaluate statistical differences between the two groups. **p < 0.01, ***p < 0.001, ****p < 0.0001. Created with GraphPad Prism.

Across the 115,171 unique variants, 2.57% had DG scores ≥0.2 at DPs <3 and 0.61% had DG scores ≥0.8 at DPs <3 (Fig. 2A). Findings were similar across the three datasets (Fig. 2A). As expected (Fig. 1), nonsense variants with DG scores ≥0.2 at DPs <3 were significantly more likely to be TAA or TGA stop codons (63.1%) than the remaining nonsense variants in the overall dataset (56.9%; chi-square = 59.5, *p* < 0.00001). The proportion of nonsense variants that were TAA or TGA stop codons increased to 72.1% when restricting to the subset meeting the high precision threshold of DG scores ≥0.8. Also as expected (Fig. 1), the predicted new donor splice sites clustered at the -2 pre-mRNA position (Supplemental Fig. 1).

We then investigated whether this molecular mechanism could explain some instances of apparent incomplete penetrance and highly variable expression. Restricting to the ClinVar dataset, nonsense variants potentially triggering a manufactured splice rescue were significantly less likely than the remaining nonsense variants in the same genes to have a likely pathogenic or pathogenic (LP/P) classification (Fig. 2B). There were also differences in the confidence level (star rating) of those LP/P classifications (Fig. 2C), including a lower mean star rating in nonsense variants potentially triggering a manufactured splice rescue compared with the remaining nonsense variants in the same genes (SpliceAI ≥ 0.2: 1.10 vs. 1.34, respectively, W = 4944247, *p* < 0.00001; SpliceAI ≥ 0.8: 1.11 vs. 1.36, W = 584088, *p* < 0.0001).

**Fig. 3:**
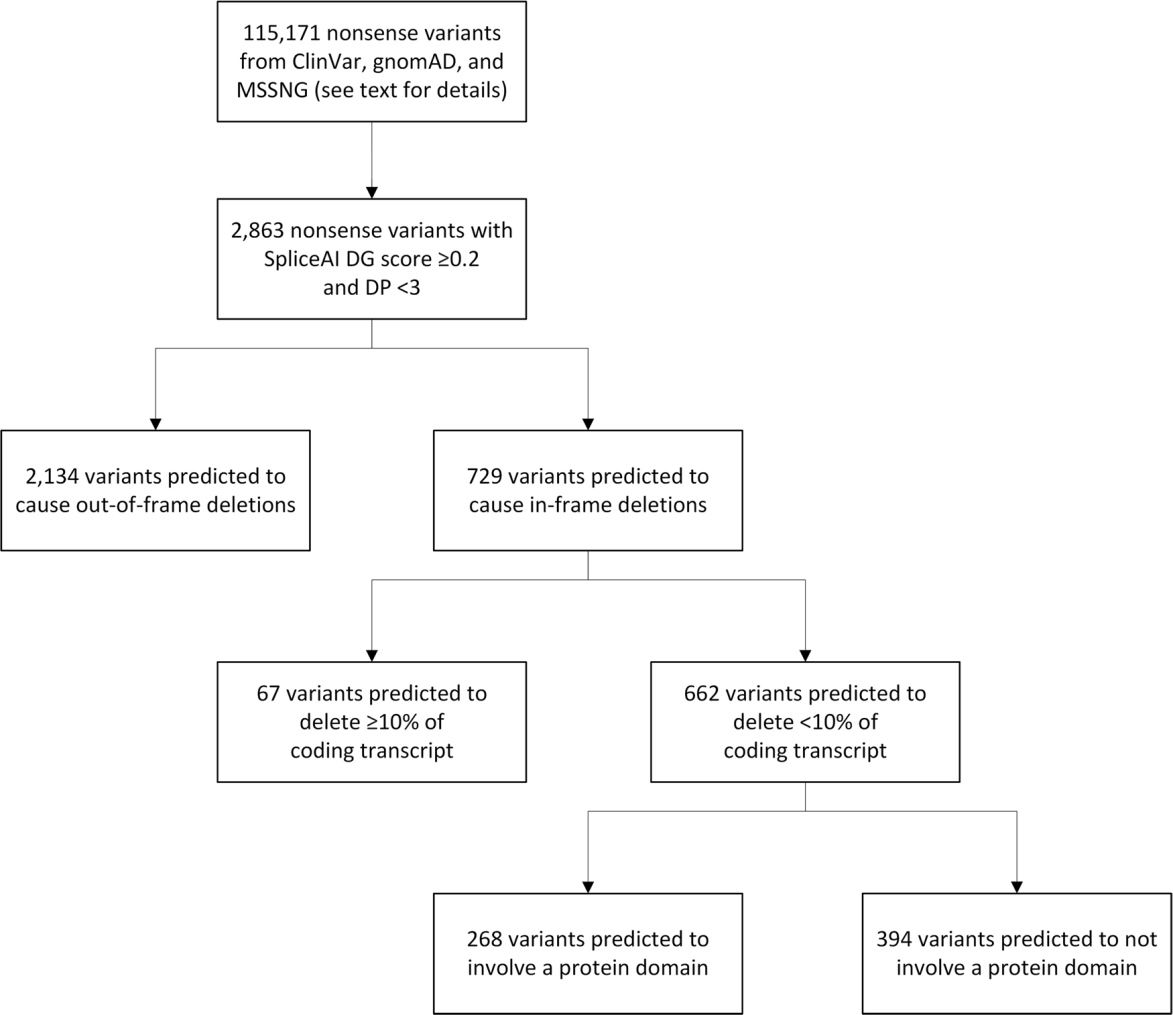
In silico predictions of protein-level consequences indicate that a proportion of all nonsense variants may evade loss-of-function through “manufactured splice rescue”. See text for details. With the assumption that creation and use of a new donor splice site within an exon will lead to deletion of the downstream component of that exon (Fig. 1), variants were categorized as causing in-frame deletions or out-of-frame deletions, then as deleting <10% or ≥10% of the coding transcript, and then as to whether the deletion did not or did involve a protein domain. DG donor gain, DP delta position.

Considering the subset of nonsense variants that met our SpliceAI cut-offs of DG delta score ≥0.2 at DPs <3 (*n* = 2863), and assuming partial exon deletion as a result of using the newly created donor splice site (Fig. 1), we predicted that 662 nonsense variants (23.1% of 2863, or ~1 in 175 of all 115,171 nonsense variants) would result in in-frame deletions accounting for <10% of the coding transcript (Fig. 3). There was a non-significant trend towards nonsense variants predicted to result in in-frame deletions being less likely than nonsense variants predicted to result in out-of-frame deletions to be classified as LP/P variants in ClinVar (87.6% vs. 90.7%, *p* = 0.78). A proportion of the variants also impacted protein domains (Fig. 3), however whether small in-frame deletions in these domains would disrupt overall protein function could not be determined.

For example, a nonsense variant in *TSC2* (NM_000548.5:c.4081C > T) was reported in ClinVar (SCV000819981.3) as a variant of uncertain significance after it was identified in an individual without features of tuberous sclerosis complex. This variant’s SpliceAI DG score is 0.80, and additional in silico tools also predict the creation of a donor splice site 2 bp upstream to the variant position in the pre-mRNA (Supplemental Fig. 2). In silico analysis of the variant suggests the outcome may be an in-frame deletion [GRCh38(Chr16):g.2084302_2084950del; p.(Glu1360_Ser1498delinsAsp)] that removes <10% of the total protein length and does not impact key functional protein domains.

## Discussion

Secondary sequence properties can alter the predicted impacts of variants [15]. However, consideration of “manufactured splice rescue” (in contrast to other mechanisms, like “naturally occurring candidate rescue transcripts” [7]) is not yet codified in variant classification criteria for nonsense variants [1, 2, 7]. We found only rare instances of it being acknowledged by clinical genetic testing laboratories during variant review (e.g., ClinVar Accession: SCV002216056.2). Inspired by a recent case report [3], we found evidence that this molecular mechanism could apply to a small but meaningful proportion of all nonsense variants.

Our preliminary study has several limitations. In silico prediction scores are imperfect [11, 16]. We did not confirm the splicing effect of specific nonsense variants in individuals by RNA sequencing [3, 13, 17] or other functional assays [18]. The creation of a splice site upstream of the nonsense variant might still result in a loss-of-function (e.g., from an indel that results in a frameshift). The predicted impact of an in-frame deletion within a protein domain on protein function is best determined on a gene-by-gene basis through manual curation of the literature and/or experimental (in vivo or ex vivo) approaches, and was beyond the scope of this report. Conversely, nonsense variants may be rescued by different mechanisms unrelated to manufactured splice rescue [6, 7, 15, 19, 20]. Lastly, while we explored three different datasets (ClinVar, gnomAD, and MSSNG) to offset the ascertainment biases inherent in each and noted similar expected rates of manufactured splice rescue, none provides an unbiased sampling of germline human nonsense variants. The true prevalence in the genome of this phenomenon of manufactured splice rescue remains unknown.

In summary, we have assessed an underappreciated mechanism whereby unchallenged assumptions regarding variant impact could result in inaccurate variant interpretation. There is growing awareness that in silico tools like SpliceAI are invaluable for identifying deleterious cryptic splice variants within classes of variation often presumed to be benign (e.g., synonymous variants, deep intronic variants) [16], but the inverse scenario is rarely considered. We recommend against initially applying PVS1-level evidence to novel nonsense variants where manufactured splice rescue is a strong possibility and correlation with phenotype is challenging, as will often be the case with secondary findings and in the anticipated future wave of newborn genomic screening programs.

### Supplementary information

Supplemental file

## Data Availability

The datasets analysed during the current study are available in the ClinVar [ncbi.nlm.nih.gov/clinvar/], gnomAD [gnomad.broadinstitute.org/], and MSSNG [research.mss.ng/] repositories.
