# Supplementary material for: Estimating the proportion of nonsense variants undergoing the newly described phenomenon of manufactured splice rescue
Source: Eur J Hum Genet. 2023 Nov 27;32(2):238–42. doi: 10.1038/s41431-023-01495-6 (PMC10853237; doi:10.1038/s41431-023-01495-6)

Supplemental Figure 1. Pre-mRNA positions of DG splice sites with high SpliceAI scores (≥0.8) created by nonsense variants across gnomAD, MSSNG, and ClinVar.
Analyses focused on scores with pre-mRNA positions <3 (i.e., excluding splicing changes downstream of the resulting stop codon). See text for details.

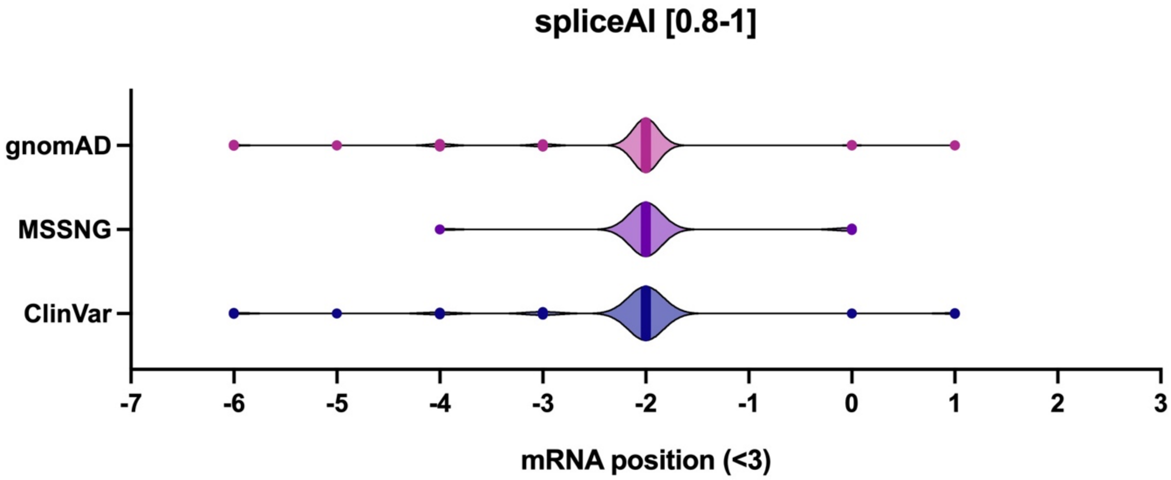

Supplemental Figure 2. Visualization of a 37 bp window of the MANE Select *TSC2* transcript containing a C>T bp change at cDNA position 4081.
The top strand represents the reference sequence and the bottom strand represented the variant sequence. Highlighted nucleotides indicate the bp change. Blue markers indicate *in silico* prediction scores from SpliceSiteFinder-like, MaxEntScan, NNSPLICE, and GeneSplicer for a donor splice site. See text for details. Image produce by Alamut Visual Plus (v1.7 © 2022 SOPHiA GENETICS).

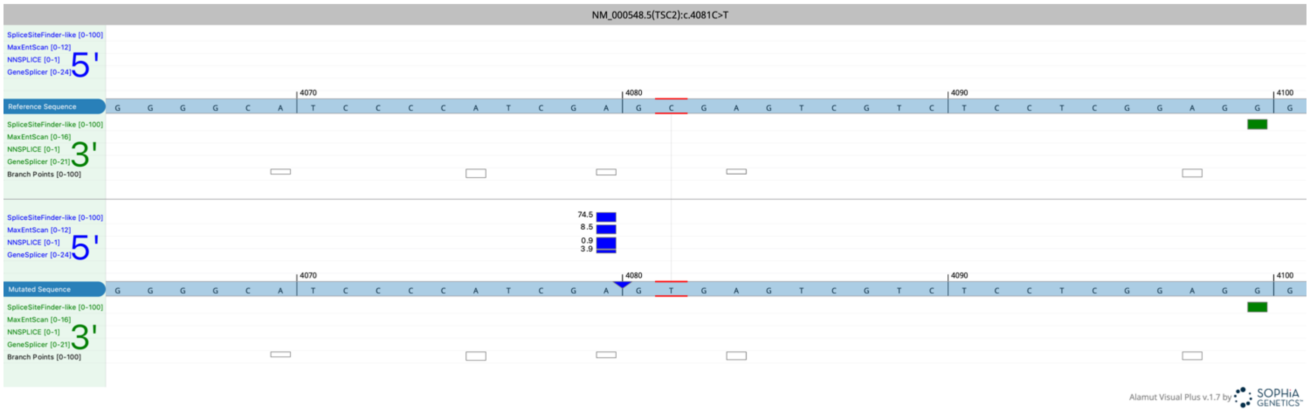

Supplement: Supplementary file 1 — Supplemental file [file 41431_2023_1495_MOESM1_ESM.pdf]
